# Incidental Chronic Neutropenia in an Asymptomatic Adult

**DOI:** 10.7759/cureus.1779

**Published:** 2017-10-17

**Authors:** Abhishek Gupta, Anurag Dhingra

**Affiliations:** 1 Geriatrics, Center for Addiction and Mental Health; 2 Family Medicine, Star Medical Center, Mississauga, On, Canada

**Keywords:** neutropenia, incidental, polyclonal gammopathy, infection risk, opportunistic, idiopathic, asymptomatic

## Abstract

Chronic neutropenia is a rare hematologic abnormality encountered in primary care. It can be caused by a wide range of acquired and congenital factors. Very rarely, it can occur as isolated chronic neutropenia where other hematologic cell lines are completely intact. The case discussed here dealt with a similar situation where a 29-year-old female patient presented with severe neutropenia and otherwise intact cell lines in an asymptomatic fashion. Laboratory testing conducted at multiple intervals showed a consistently and severely depressed absolute neutrophil count (ANC) for a prolonged time. In addition, the patient had some abnormalities in serum immunoglobulin levels that pointed towards an underlying autoimmune or malignant pathology but these were insufficient to arrive at a clear diagnosis. The unique presentation in this patient presents an opportunity to study the pathological causation for neutropenia and, more specifically, isolated neutropenia.

## Introduction

Hematologic and rheumatologic abnormalities such as chronic idiopathic neutropenia are rarely observed in primary care. Often, the standard protocol for such encounters is to seek consultations with hematologists and rheumatologists. However, the same patient's general healthcare is an especially challenging situation for the primary caregiver itself. This report describes a similar case where a particularly severe case of neutropenia was managed in an outpatient setting by a family physician. Such a scenario creates a severe risk of infections due to the associated lack of immunity, raising important questions regarding specific criteria for the administration of prophylactic medications and hospital admission. The asymptomatic nature of the patient's condition required a primarily outpatient management course. While there are established guidelines for the inpatient management of such situations, this case will highlight the lack of appropriate guidelines for primary care providers for such exotic conditions.

## Case presentation

The patient is a 29-year-old female of Asian Indian origin who presented for a routine annual screening. Her family history was insignificant for hematologic or rheumatologic disorders. Her personal medical history included travel to India two months prior to the initial presentation, where she stated that she experienced a urinary tract infection (UTI) and was successfully treated with nitrofurantoin (unknown dose and regimen) in an outpatient setting. Other significant events in her history included a distant episode of typhoid, successfully treated at an unknown time. Her symptoms at the time of the initial visit included mild fatigue in daily activities without muscle stiffness or rigidity. A thorough history and physical exam were conducted, especially to rule out abnormalities in the endocrine aspect. However, these preliminary investigations were negative for any significant abnormalities. The patient denied having any environmental/medical allergies and ingesting any medication or nutritional supplements.

Prior hematologic profiles for this patient, obtained from prior medical providers, indicated a significant history of low absolute neutrophil count (ANC) at multiple intervals (four to five years prior), ranging from 0.8-1.5 (x 10*9/L) (*n*: 2-8 x 10**9/L). However, the patient could not identify any significant medical events or illness in relation to the same lab abnormalities in her history.

At her first visit, her overall white blood cell count (WBC) was depressed (2.8 x 10**9/L) (normal range (*n*): 4-10 x 10**9/L) as was her ANC (0.5 x10**9/L) (*n*: 2-8 x 10**9/L). In addition, her red blood cell distribution width (RDW) was elevated (15.2%) (*n*: 11.5-14.5%), low high-density lipoprotein (HDL) (0.54 mmol/L) (*n*: 40-80 mmol/L) and her vitamin B12 was marginally low (127 pmol/L) (*n*: 130-700 pmol/L). Her serum studies were negative for any abnormalities in electrolytes, thyroid stimulating hormone (TSH), other hematologic cell lines (including lymphocytes, basophils, monocytes, and eosinophils), ferritin, hemoglobin (Hb), hematocrit (Hct), mean corpuscular hemoglobin concentration (MCHC), renal function, fasting glucose, low-density lipoprotein (LDL), red blood cells, folate, and follicle stimulating hormone (FSH)/luteinizing hormone (LH). See Table 1.

**Table 1:** Periodic Hematologic Measurements in a Patient with Idiopathic Neutropenia All values are in x10**9/L.

Date	Total WBC	Neutrophil	Lymphocyte	Monocyte	Eosinophil	Basophil
Normal reference range	4 - 10	2 - 7	1 - 3	0.2 - 1.0	0.02 - 0.5	0.02-0.1
27/02/2016	2.8	0.5	1.8	0.4	0.1	0.0
03/03/2016	3.3	0.7	2.2	0.4	0.1	0.0
20/05/2016	3.0	0.5	2.1	0.4	0.0	0.0
30/05/2016	2.6	0.4	1.8	0.4	0.1	0.0
07/06/2016	3.4	0.6	2.1	0.6	0.1	0.0
24/06/2016	2.5	0.2	1.8	0.4	0.1	0.0

Follow-up visits

Initial impressions suggested that the patient's neutropenia may be a result of an underlying vitamin deficiency. As such, the patient was immediately advised to begin an oral regimen of over-the-counter (OTC) multivitamin supplements, including Vitamin B12, and to report to a medical facility upon any febrile or upper respiratory infection symptoms. In addition, serologic testing for human immunodeficiency virus (HIV) and Epstein-Barr virus (EBV) was conducted and found negative. Also, it was suspected that the vitamin deficiencies may have been due to errors in the initial lab testing of collected samples. Therefore, a repeat serum vitamin folate/B12 measurement panel was performed, which showed precisely the same results as previously reported (a borderline low serum vitamin B12 level and normal serum folate level).

Regular and periodic monitoring of serum vitamin B12/folate levels was a significant part of the following investigation. This was due to the initial hypothesis that the patient's underlying neutropenia and mild fatigue were a subclinical presentation of an underlying vitamin deficiency. Consequently, as mentioned previously, OTC vitamin supplementation was recommended and the same serum vitamin levels were monitored. Results showed consistently normal levels of folate along with a declining serum vitamin B12 (110 pmoL/L) (*n*: 130-700pmol/L) level. During these follow-up visits, it should be noted that the patient admitted to not being fully compliant with the previous recommendation for daily multivitamin supplementation.

Subsequent investigations also focused on the common rheumatologic, hepatic, and hematologic serum markers as well as on tracking the serum neutrophil count and screening for any opportunistic infections. Apart from the abnormal vitamin B12 level, an elevated erythrocyte sedimentation rate (ESR) (80 mm/H) (*n*: 0-29 mm/hr) was also observed. In addition, the physical examinations at routine follow-up intervals focused on screening for febrile symptoms or any other symptoms associated with rheumatologic conditions [1]. Splenomegaly was an especially important part of the physical exams during follow-up visits, as it is a significantly associated physical symptom with hematologic deficiencies, rheumatologic abnormalities, and infections [1].

Further testing showed elevations in serum IgG (19.39 g/L) (*n*: 6.2-14 g/L) and IgA (4.97 g/L) (*n*: 0.8-3.5 g/L), while serum protein electrophoresis abnormalities included elevations in beta-2 (6.2 g/L) (*n:* 0.7- 1.5 g/L) and G-globulin (17.9 g/L) (*n*: 0.5-1.4 g/L). However, urinalysis for Bence Jones proteins and monoclonal light chains and serum testing for anti-nuclear antibodies, alanine/aspartate aminotransferase, tuberculosis, and IgM elevation were negative as well. This polyclonal gammopathy indicated an underlying state of inflammation and, therefore, the patient was referred to a hematologist and a rheumatologist, which also confirmed the persistent neutropenia but could identify the cause of the same inflammation.

The considerably low ANC warranted a bone marrow biopsy, which the patient agreed to undergo but at a later, unspecified time. However, given the asymptomatic nature of the patient's presentation, the patient did express hesitance for undergoing the same biopsy. Therefore, despite counseling the patient to undergo the biopsy, the patient indefinitely postponed the procedure.

## Discussion

Neutropenia is defined as an absolute neutrophil count less than 1.5 x10*9/L, with chronic neutropenia classified if the abnormality persists for more than three months [2]. Such chronic neutropenia is classified as severe when less than 0.5 x10*9/L [2]. While this can occur incidentally in persons with good health, a persistent pattern requires thorough investigation to identify the underlying cause [2].^ ^The most common causes of chronic neutropenia are listed in Table 2.

**Table 2:** Causes of Chronic Neutropenia Table adapted from "Evaluation and Management of Patients with Isolated Neutropenia" by Newburger PE and David CD, 2013, Seminars in Hematology, Vol. 50, Pg 199 [2]

Extrinsic	
Nutritional	Vitamin B12, folate, copper, protein-calorie deficiencies
Immune	Autoimmune
	Congenital immunologic disorder
	Systemic autoimmune disorder (for example, rheumatoid arthritis, systemic lupus erythematosus, Felty's syndrome)
Intrinsic	
Myelodysplasia	
Acquired bone marrow failure	Aplastic anemia
Congenital bone marrow failure	
Isolated neutropenia	Severe congenital neutropenia; cyclic neutropenia
Neutropenic syndromes	Disorders of granule sorting: Chediak Higashi syndrome, Cohen syndrome, Griscelli syndrome type 2, Hermansky-Pudlak syndrome type 2, and p14 deficiency
	Metabolic disorders: glycogen storage disorders type 1b, Barth syndrome, Pearson syndrome
	Disorders of immune function: hyper-IgM (Jobs') syndrome, warts, hypogammaglobulinemia, infections, and myelokathexis (WHIM) syndrome; cartilage-hair hypoplasia; Schimke immune-osseous dysplasia
	Disorders of molecular homeostasis: dyskeratosis congenita, Fanconi syndrome, Shwachman-Diamond syndrome

Chronic management of this case involved a dual and systematic approach. One aspect of the approach involved screening for any symptoms associated with opportunistic infections and rheumatologic conditions involving neutropenia such as rheumatoid arthritis, systemic lupus erythematosus, or Felty's syndrome [2]. As previously stated, splenomegaly was one of the symptoms that were screened for. However, the screening regimen did not involve any imaging steps to identify accessory splenic tissue, also possibly associated with rheumatologic conditions [2]. The second aspect focused on a laboratory-based analysis and a monitoring of hematologic as well as rheumatologic markers, including ESR and C-reactive protein (CRP).

Commonly, any case of neutropenia in an adult individual involves a step-by-step approach, which was adopted here [3]. Any febrile cases or any individual with an ANC less than 0.2x10*9/L require immediate hospitalization along with a broach spectrum antibiotic course with inpatient observation. Anaerobic coverage is also required if abdominal pain is present in addition to fever [2]. This patient previously underwent two hospitalizations with subsequent discharge as a preventative measure due to extremely low ANC (0.2 x 10*9/L). While the hospitalizations were recommended by a prior medical provider, who was unable to be contacted, the hospitalization records and the patient herself noted the reasoning to be the severe neutropenia itself, given that the ANC was within borderline-low levels to justify the hospitalization.

The second step involves an identification of whether this episode is a new onset or a new episode in recurrent neutropenic pathology [3]. If positive for recurrence, a high degree of suspicion is held for an ethnic, benign, familial, or cyclic neutropenia [2]. Ethnic variants of chronic neutropenia are present in African American populations or those negative for the Duffy blood group, but this patient lacked any such characteristic [2]. However, hematologic consults did have a suspicion for ethnic/normal variation in ANC due to the episodic nature of the patient's neutropenia.

Thirdly, the management process must identify recent drug or infectious exposure [3]. If positive and yet self-resolving, a definitive case for drug/infection-mediated neutropenia can be made [3]. While this patient presented with chronic neutropenia, the patient reported prior episodic neutropenia as well. However, this patient was not exposed to any such agents or infections (Table 3). It should be noted that despite this patient's exposure to nitrofurantoin, sulfa agents are typically responsible for transient neutropenia only (lasting less than 3 months) [2]. As such, this highlights the importance of screening for any factors that may cause repetitive episodic and transient neutropenia, giving the illusion of chronic neutropenia. Therefore, risk factor screening and periodic serum measurements were critical in eliminating any causes of transient neutropenia.

**Table 3:** Causes of Transient Neutropenia Table adapted from "Evaluation and Management of Patients with Isolated Neutropenia" by Newburger PE and David CD, 2013, Seminars in Hematology, Vol. 50, Pg 199 [2] HIV: human immunodeficiency virus

Infection	
Viral	Cytomegalovirus, Epstein-Barr virus, HIV, influenza, parvovirus B19
Bacterial	Brucella, paratyphoid, tuberculosis, tularemia, typhoid; Anaplasma phagocytophilum, and other rickettsia
Protozoan	Plasmodium Vivax, Plasmodium Falciparum
Drugs	
Anticonvulsant	Carbamazepine, valproate
Antimicrobial	Sulfonamides, penicillins, trimethoprim/sulfamethoxazole
Antipsychotic	Clozapine, olanzapine, phenothiazines
Anti-rheumatic	Gold, levamisole, penicillamine
Antithyroid	Methimazole, propylthiouracil
Other	Aminopyrine, deferiprone, rituximab, levamisole-adulterated cocaine
Immune	Neonatal isoimmune, autoimmune

In continuation of the third investigative step, if the neutropenia from a suspected drug or infectious agent is not self-resolving, a bone marrow biopsy/aspirate or flow cytometry is indicated. If dysplastic, myelodysplasia is the most common culprit. If normal, a diagnosis of idiopathic neutropenia can be made. However, large granular lymphocytic leukemia (LGL) usually presents with abnormal markers of flow cytometry (CD 3,8,16, 57 clonal expansion or natural killer cell clonal expansion) [2]. As such, the third step is to identify drug-based, infectious, or any dysplastic reasons for an adult patient's neutropenia [3].

The following steps focus on screening for other cytopenias and megaloblastic anemia. The former is identified by obtaining a bone marrow biopsy/aspirate or flow cytometry. The latter is identified via serum vitamin B12 and folate levels. Measuring the serum vitamin b12/folate does not necessarily have to be an end diagnostic step, as it can be performed concurrently to earlier diagnostic steps also. However, bone marrow biopsy/aspiration or flow cytometry is advised at a later stage in the diagnostic process due to the relative ease of serum-based laboratory testing [3].

This patient's management reflected the above process, which is the commonly established method of dealing with a neutropenic adult patient. The final step of the investigative process, i.e., bone marrow biopsy is indefinitely postponed for this patient until the patient consents to undergo the procedure. In terms of treatment, a neutropenic adult can be managed based on whether an underlying pathology is present or not (familial, constitutional, or cyclic neutropenia) [3]. Treatment options usually include granulocyte-colony stimulating factor (G-CSF), glucocorticoids, nutritional supplements (vitamin B12, folate), splenectomy, medication review, antibiotics, and granulocyte transfusion [4].

The recommended hygienic precautions for an asymptomatic adult neutropenic individual, such as in this case, primarily include simple soap-and-water hand-washing regimens [2]. Advanced precautions, such as sterilization of living space, are usually not necessary and have no additional benefit [2]. Further recommendations include avoiding extremely crowded areas and close contact with infected individuals to reduce hospitalization risk [2]. Additionally, opportunistic infection risk can be reduced by avoiding contaminated sources such as mulch and construction/demolition sites with dust or animal/bird waste [2]. Lastly, the risk of gingivitis or tooth loss must also be reduced via good dental hygiene [2].

G-CSF, commonly used in addressing neutropenia secondary to chemotherapy, can also be beneficial in reducing the risk of oral ulcers or febrile events/infections in cyclic, benign, or familial neutropenias [2]. However, in this patient, such treatment was deferred until a concrete diagnosis was obtained via a bone marrow biopsy and due to her asymptomatic presentation. It is also important to note that myeloid growth factor therapy is traditionally administered in individuals with a substantial history of infections or oral pathology demanding an expensive course of G-CSF [2]. As these factors were also lacking in this patient, G-CSF was currently not administered.

Alternative treatment modalities include corticosteroids, which usually have very modest improvements in neutrophil levels [2]. However, corticosteroids have a severe risk of increasing a patient's infectious susceptibility along with numerous other side-effects, which are undesirable in this case [2]. Neutrophil transfusions, another alternative treatment, is only used as a short-term measure due to the risk of alloimmunization and severe transfusion reactions [2]. Lastly, hematopoietic stem cell transplants are only used after G-CSF regimens have failed or if the patient's condition carries a high risk of evolution to myelodysplasia [2]. Due to the lack of any specific indications or symptomatic severity, the above alternative treatments were not enacted in this patient.

Despite the idiopathic and asymptomatic nature of the patient's neutropenia, the patient's management required a highly systematic and comprehensive approach. This involved assessing the immediate risk for infection (based on serum ANC) and the relative need for hospitalization while educating the patient about the necessary hygiene-related precautions. Meanwhile, the diagnostic process systematically eliminated autoimmune, nutritious, or infectious causes. Due to the patient's refusal to undergo a bone marrow biopsy, the underlying etiology of the neutropenia could not be established. Therefore, the patient was treated on an outpatient basis under the final diagnosis of chronic idiopathic neutropenia. However, the management of this patient was limited significantly by a lack of any established guidelines that addressed the asymptomatic and chronic aspects of an adult with severe neutropenia. 

## Conclusions

This participant presented a unique opportunity to study an asymptomatic and, yet, very severe case of neutropenia with polyclonal gammopathy. The diagnostic process involved an exhaustive and systematic investigation into this participant's history of exposure to drugs, infectious agents, or any symptoms associated with rheumatic conditions. Additionally, the management of this patient involved essential precautions and education against opportunistic infection risks, mainly via good hygiene maintenance and routine symptom/lab monitoring. Further care in this patient, while heavily dependent on the bone marrow biopsy result, did not dictate the entirety of the patient's management, especially in the outpatient setting. The systematic approach mentioned above was essential to minimize the risk of infections in this patient while a prolonged diagnostic process was carried out. This case highlighted the challenges involved in managing idiopathic pathologies in primary care that necessitate the establishment of clear guidelines and algorithms for exotic scenarios such as with this patient.
